# The history of hexachlorobenzene accumulation in Svalbard fjords

**DOI:** 10.1007/s10661-018-6722-3

**Published:** 2018-05-24

**Authors:** A. Pouch, A. Zaborska, K. Pazdro

**Affiliations:** Institute of Oceanology Polish Academy of Sciences, Powstańców Warszawy 55, 81-712 Sopot, Poland

**Keywords:** Hexachlorobenzene, Persistent organic pollutants, Sediment, Svalbard fjords

## Abstract

In the present study, we investigated the spatial and historical trends of hexachlorobenzene (HCB) contamination in dated sediments of three Svalbard fjords (Kongsfjorden, Hornsund, Adventfjorden) differing in environmental conditions and human impact. HCB concentrations ranging from below limit of quantification (6.86 pg/g d.w.) to 143.99 pg/g d.w. were measured. The highest concentrations were measured in two surface sediment layers of the core collected in Hornsund near the melting glacier. The lowest concentrations of HCB were measured in Adventfjorden, suggesting that local source of HCB is not significant and global transport processes are the major transport pathways. The history of HCB deposition did not fully reflect the history of HCB emission (largest in 1950s and 1960s). In case of several sediment cores, the HCB enrichment in surface (recent) sediments was noticed. This can indicate importance of secondary sources of HCB, e.g., the influx of HCB accumulated over decades on the surface of glaciers. Detected levels of HCB were generally low and did not exceed background concentration levels; thus, a negative effect on benthic organisms is not expected.

**Electronic supplementary material:**

The online version of this article (10.1007/s10661-018-6722-3) contains supplementary material, which is available to authorized users.

## Introduction

Hexachlorobenzene (HCB) is an organochlorine compound that has been produced since 1933 (Barber et al. 2005). It was widely used in the 1940s and 1950s as a fungicidal wood-preserving agent and to control wheat bunt (Cripps et al. 1980; Gocmen et al. 1986). At its peak, global HCB emissions have been estimated at up to 92,000 kg/year (Bailey 2001). HCB has also been used as a porosity control agent in the manufacture of graphite anodes, as a peptizing agent in the production of nitroso and styrene rubber for tyres (Carpenter et al. 1986; Mumma and Lawless 1975) and in the production of pyrotechnics for the US and Russian military (Shekhovtsov 2002). In the late 1960s, HCB was recognized to have negative effects on non-target organisms, inter alia to cause a suppression of, or decrease in reproduction and to weaken immune system. Its use was thus restricted in many countries (AMAP 2004; Barber et al. 2005). HCB is considered as one of the 12 most persistent organic pollutants and was included in the Stockholm Convention (2001) as a pollutant requiring an action plan. It is also listed as priority chemicals within the EU Water Framework Directive (WFD). Although production and use of HCB has ceased in most countries, it is still emitted to the environment as a by-product of the production of several pesticides which contain chlorine, the manufacture of chlorinated solvents, the production of certain industrial chemicals and the combustion of waste and materials containing chlorine (Barber et al. 2005; UNEP 2005; Voldner and Smith 1989). Current world emissions are estimated to be 70–95% lower than in the 1960s (Wang et al. 2010). Moreover, significant re-emission of “old” HCB from soils and sediments may occur (Barber et al. 2005). HCB, which has an assigned approximate half-life of 2 years in the air and 6 years in water and sediments, is thought to be highly persistent in the environment (Mackay et al. 1992). HCB degradation processes occur slowly, and it can be degraded by photolysis (Schauerte et al. 1982) and/or microbial activity (Beurskens et al. 1993).

HCB is a highly volatile contaminant and therefore is transported even to remote locations (Wania and Mackay 1996). It can then be removed from air by wet and dry atmospheric deposition to water and land surfaces. From marine waters, some HCB may be retransferred into the atmosphere by volatilization, while some may be adsorbed onto organic matter and very fine mineral particles and sink to the sea bed.

The Arctic is believed to be a sink for contaminants emitted in the northern hemisphere (Macdonald et al. 2005). It receives contaminants mainly from long-range atmospheric transport, oceanic transport, and drifting sea ice (Macdonald et al. 2005). For volatile organic contaminants, air circulation is the most important transport pathway. Organic contaminant transport occurs particularly during winter and spring, when the cold condensation process removes contaminants from the atmosphere (Wania and Mackay 1996).

There are few studies of HCB concentrations in the Arctic environment, and most of them concern the atmosphere (e.g., Wu et al. 2014; Jaward et al. 2005; Lammel et al. 2007). Since 1990, HCB concentrations in the air have been measured by the Norwegian Institute for Air Research (NILU) in Ny-Ålesund (Kongsfjorden, Svalbard) at Zeppelin station, and data are available on the internet website (http://www.nilu.no/projects/ccc/onlinedata/pops/index.html; NILU 2016). HCB concentration in the air has been generally constant since 1990 and accounts for ~ 4.5 pg/m^3^, with seasonal increases to 7 pg/m^3^. Higher concentrations are attributed to evaporation of HCB accumulated previously on surfaces of sea ice and glaciers (Hung et al. 2010). The observed air concentrations do not show a declining trend, which is probably a result of emission from secondary sources and continuous use of pesticides containing HCB in some countries (Ambrus et al. 2003). According to Cai et al. (2012), the concentrations of dissolved HCB in the seawater increase with increasing latitudes to 7.1 pg/dm^3^ above 73°N, confirming the major role of air cold condensation in long-range transport of organic pollutants. The concentrations of HCB in marine components of the Arctic fjords have been poorly studied, though the concentration of HCB in sub-surface layers of seawater of Kongsfjorden and Liefdefjorden has been determined at 4.9–5.2 pg/dm^3^ (Hallanger et al. 2011).

Knowledge of the distribution of HCB within sediments is important, as benthic organisms living on the sea bed are constantly exposed to contamination. HCB can be accumulated by marine organisms through direct contact with water, by ventilation, or by ingestion. Since HCB is lipophilic (log *K*_ow_ = 5.5), it bioaccumulates easily and biomagnifies in the food web (Euro Chlor 2002). Exposure to HCB causes malfunctioning of reproduction and immune systems (AMAP 2004). The predicted no effect concentration value for chronic exposure (PNECchronic) is defined as 17 ng/g for HCB in sediments (SFT 2007a,b). Long-term exposure to sedimentary HCB concentrations above these levels can cause toxic effects. HCB bioconcentration factors range from 375 to > 35,000 depending on organism species (WHO 1998). The average biomagnification factor for HCB in the food web of Baffin Bay and the Barents Sea is 4.1 (AMAP 2004).

A better understanding of the organic contaminant distribution within fjord environments is also very important since global changes are affecting the strength and directions of contaminant transport processes (Macdonald et al. 2005). Due to melting of glaciers, ice cover, and permafrost, HCB accumulated on, for example, glacier surfaces for many decades, can be discharged to marine waters. Since sediments are the final sink for most contaminants, the history of Arctic contamination can be retrieved by investigation of historical contaminant distributions within sediments. The objective of this study was to investigate the spatial and historical trends of HCB contamination in dated sediments of three Svalbard fjords differing in environmental conditions and human impact. The potential toxicological impact of HCB on the benthic community is discussed.

## Materials and methods

### The study area

**Fig. 1:**
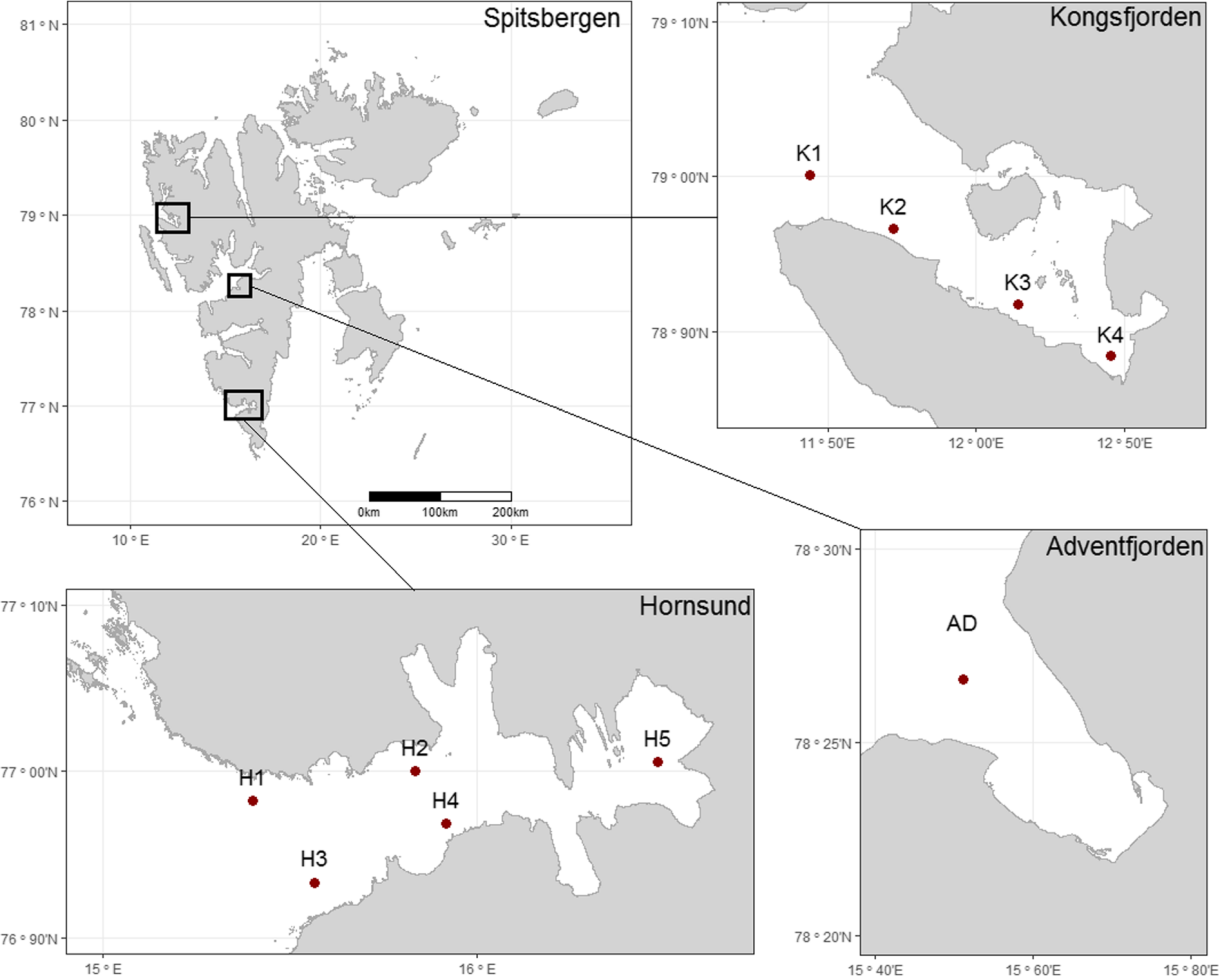
Location of sampling stations in Kongsfjorden (K1–K4), Hornsund (H1–H5) and Adventfjorden (AD)

The Spitsbergen, the largest island Svalbard archipelago, is located from 74° to 81° N (Fig. 1). Fjords selected for the study are located on the western part of the island and are characterized by different environmental conditions. Kongsfjorden located in the north part of Spitsbergen is a 20-km-long fjord divided into two parts: inner basin of 50–60-m average depths and outer basin with average depths of 200–300 m. The outer part of the fjord is influenced by warm Atlantic waters of West Spitsbergen Current (WSC; Svendsen et al. 2002). The inner part hosts five tidewater glaciers providing the major sources of freshwater. The mean annual freshwater input is about 1.4 km^3^ and constitutes about 5% of the mass balance in the fjord (Cottier 2005; Svendsen et al. 2002). Hornsund located in the southern part of the island is a 34-km-long fjord. The outer and central parts of the fjord (100–260 m) are affected by Arctic waters transported by Sørkapp Current and by Atlantic waters of WSC (Drewnik et al. 2016). The shallower inner part—Brepollen—dominated by locally produced Arctic waters rarely receives inputs of warm waters of Atlantic origin. About 67% of Hornsund area is covered by glaciers, particularly Brepollen receives large amount (1.8 km^3^) of freshwater from two melting glaciers Hornsbreen and Svalisbreen (Błaszczyk et al. 2013).

Additionally, to assess the impact of local sources of pollution, sediment cores were collected from Adventfjorden (located near Longyearbyen town, the largest Svalbard settlement). The possible contaminant sources from Longyearbyen include previous and recent coal mining, energy production, transport, municipal wastewater, and solid waste storage and combustion. Untreated municipal wastewater is discharged directly to the fjord waters (Olsson 2016). Adventfjorden, the southern branch of Isfjorden, receives a mixture of WSC and arctic waters and also a large input of freshwater from a snow cover and sea ice melts from April to June and glacial river (Węsławski 2011).

### Field sampling

Sediment cores were collected in 2007, 2013, and 2014, from the r/v Oceania at 10 stations located in different parts of the fjords (Fig. 1, Table 1). Five stations were sampled in Hornsund (H1, H2, H3, H4, H5), four in Kongsfjorden (K1, K2, K3, K4), and one in Adventfjorden (AD). The 20–40-cm-long sediment cores were collected using a Gemax corer or a Niemistö corer. After collection, sediment cores were sliced at 1-cm intervals (layers 0–10 cm) and 2-cm intervals (layers 10–40 cm) and frozen immediately. Prepared samples were transported and analyzed in the laboratory of the Marine Chemistry Department, Institute of Oceanology, Polish Academy of Sciences.

**Table 1:** Description of sampling stations in three Svalbard fjords (Kongsfjorden, Hornsund, and Adventfjorden), including coordinates, water depth, sediment accumulation rates (Zaborska et al. 2017), organic matter content (*M*_org_ content), and fine fraction (Pouch et al. 2017)

Station	Coordinates	Sampling year	Water depth (m)	Sediment accumulation rates (mm/year)	*M*_org_ content (%)	Fine fraction (< 63 μm) (%)
K1	79° 00′ 03″ N 11° 26′ 17″ E	2014	330	0.22	9.9–11.8	88.6–97.4
K2	78° 57′ 58″ N 11° 43′ 11″ E	2014	296	0.28	7.9–10.4	91.9–96.6
K3	78° 55′ 03″ N 12° 08′ 31″ E	2013	96	0.47	5.3–9.1	93.2–97.5
K4	78° 53′ 02″ N 12° 27′ 07″ E	2013	84	–	5.2–7.8	97.5–99.5
AD	78° 15′ 58″ N 15° 30′ 40″ E	2013	102	0.27	6.0–7.2	81.6–93.9
H1	76° 58′ 57″ N 15° 23′ 59″ E	2007	102	0.18	4.5–7.6	91.2–96.6
H2	77° 00′ 00″ N 15° 50′ 00″ E	2007	178	0.36	8.2–11.0	94.8–98.7
H3	76° 55′ 59″ N 15° 33′ 51″ E	2007	136	0.18	5.8–7.8	95.0–96.5
H4	76° 58′ 07″ N 15° 54′ 56″ E	2007	206	0.40	4.4–9.5	83.8–97.4
H5	77° 00′ 20″ N 16° 28′ 52″ E	2013	130	0.69	3.2–8.9	93.1–93.0

### Analytical procedures

#### Preparation and sediment characterization

The sediment samples were freeze-dried (Christ Beta A apparatus), homogenized, and split into two sub-samples. The organic matter amount was calculated as a weight loss on ignition method. Sediment samples were weight before and after combustion for 5 h at 550 °C (Maher 1998). Wet sieving was used to determine the pelite fraction content (Guy 1969). The ^210^Pb measurement method (daughter nuclide —^210^Po) was used to estimate sediment accumulation rates and sediment layer ages. Sediment samples were spiked with ^209^Po (internal tracer) and digested. ^210^Po and ^209^Po activity concentrations were analyzed in alpha spectrometer (Canberra). Quality control based on measurements of reference standards (IAEA-326). The ^210^Pb_supp_ (^226^Ra) activity concentration was calculated as the average of several constant ^210^Pb values in deepest sediment layers (when possible) or/and was controlled by measurement of ^226^Ra in selected sediment layers in a high-purity germanium detector (Canberra), with 40% relative efficiency, for 3–4 days. ^137^Cs time marker has been used to control sediment accumulation rates derived from ^210^Pb dating.

#### Determination of HCB

All solvents (Suprasolv®, GC grade) and 30% Suprapur® HCl were purchased from Merck (Germany). High-purity hexachlorobenzene (99.0%) and octachloronaphthalene (99.9%) was supplied by Supelco (USA).

HCB was analyzed according to Pazdro (2007). A sample of 5 g of freeze-dried sediments was first treated by triplicate extraction with methylene chloride in ultrasonic bath. The extracts were concentrated; elemental sulfur was removed from the extract using copper powder activated with hydrochloric acid. Subsequently, samples were clean up and fractionation by absorption chromatography on silica gel (particle size 63–200 μm; Merck, Germany) and aluminum oxide (particle size 63–200 μm; Merck, Germany). Hexane was used to extract HCB. Selected samples (every fifth sample) were performed in triplicate. A gas chromatograph (Shimadzu GC 17A) with electron capture detector (ECD) was used for qualitative and quantitative analysis. The conditions of the chromatographic analysis were described in Pazdro (2007) and are presented in Table 2. An internal standard (octachloronaphthalene) was added to each sample prior to extraction to measure the recovery efficiency of the entire process. Identification of compound based on its retention time using internal and external standards. The identification was confirmed by the analysis of selected extracts by GC-MS. The five-point calibration curves in the linear range of the detector’s response were used to calculate concentration of the HCB. To monitor interferences, a procedural blank analysis was performed with every five samples. Procedural blanks were used to calculate detection limits ($LOD={\overline{x}}_{blank}+6\cdot {SD}_{blank}$) and limits of quantification (*LOQ* = 3 ⋅ *LOD*). All data were corrected for blank values. In order to test the accuracy of the quantification method, the entire analytical procedure was applied several times to a certified marine sediment (IAEA-383). The limits of quantification were 6.86 pg/g d.w. Recoveries of HCB were of 80 ± 5%.

**Table 2:** Conditions of the chromatographic analysis

Detector	Injector temperature	Detector temperature	Column	Carrier gas	Oven temperature programmer
Electron capture detector	280 °C	320 °C	DB-5 60 m × 0.25 mm × 0.25 μm	Helium, flow 1.5 ml He/min	100 °C held for 1 min; 6 °C/min to 140 °C; 2.5 °C/min to 250 °C; 10 °C/min to 310 °C, held for 20 min

### Data analysis

The linear sediment accumulation rate (cm/year) was calculated assuming an exponential decrease of ^210^Pb_ex_ with porosity-corrected sediment depth using the following equations:

$$ {\displaystyle \begin{array}{c}{A}_t={A}_0{e}^{\frac{-\lambda x}{v}}\\ {}{\ln}^{210}{\mathrm{Pb}}_{\mathrm{ex}}(x){=\ln}^{210}{\mathrm{Pb}}_{\mathrm{ex}}(0)\hbox{--} \left(\lambda /v\right)x\end{array}} $$

where ^210^Pb_ex_(*x*) is activity at layer *x*, ^210^Pb_ex_ (0) is activity at layer 0, *λ* is the decay constant, and *v* is the sediment accumulation rate.

To calculate the HCB deposition rates (ng/m^2^/yr), the HCB concentration in each sediment layer was multiplied by the mass sediment accumulation rate (presented in Zaborska et al. 2017). The HCB deposition rate calculation better illustrates the spatial differences in contaminant deposition, since its calculation takes into account the differences in sediment accumulation rates between the studied regions.

The half-life (*t*_1/2_) of HCB in sediments was calculated in order to assess the time necessary for a 50% reduction in contaminant concentration (Assefa et al. 2014; Sobek et al. 2015). It was calculated using the following equations:

$$ {\displaystyle \begin{array}{c}C={C}_0{e}^{- kt}\\ {}{t}_{\frac{1}{2}}=\ln \frac{2}{k}\end{array}} $$

where *C* and *C*_0_ are the concentrations of HCB in the surface sediment layer and the peak concentration layer (pg/g d.w.), respectively, *k* is the rate constant (year^−1^), and *t* is time in years.

The statistical analysis program, STATISTICA 10, was used to find correlations between concentrations of HCB and sediment properties (water content, organic matter, and fraction < 63 μm). The non-parametric Spearman correlation was applied to the database of obtained results. Statistical significance was accepted at *p* < 0.05.

## Results and discussion

Concentrations of organic matter ranged from 3.2 to 8.9% and were lowest in the near vicinity of the glaciers (stations K4, H5) (Pouch et al. 2017). Pelite fraction content ranged from 93 to 98% (Pouch et al. 2017). ^210^Pb activity concentration profiles for sediment cores collected from Kongsfjorden (K1, K2, K3, K4), Adventfjorden (AD), and station H5 are reported in Zaborska et al. (2017). Sediment dating was also performed for additional four stations (H1, H2, H3, H4) sampled within this study. ^210^Pb activity concentration profiles prepared for all studied stations are included in the supplement material (Fig. S1). Total ^210^Pb activity concentration in the uppermost sediments of stations H1, H2, H3, and H4 ranged from 128.2 Bq/kg in the outer part of the fjord (H1) to 170.5 Bq/kg in the central part of the fjord (H4) (Fig. 1). The mean ^210^Pb_supp_ (^226^Ra) activity concentration was of 30 Bq/kg. The accumulation rates ranged from 0.18 to 0.69 mm/year, with lower accumulation rates being determined at the outer fjord stations (H1, H3, and K1) while higher rates were found at inner fjord stations (H5 and K3). At station K4, near the Kongsbreen glacier, the sediment accumulation rate was too large to be obtained by the ^210^Pb method (Zaborska et al. 2017).

### Spatial HCB distribution

**Fig. 2:**
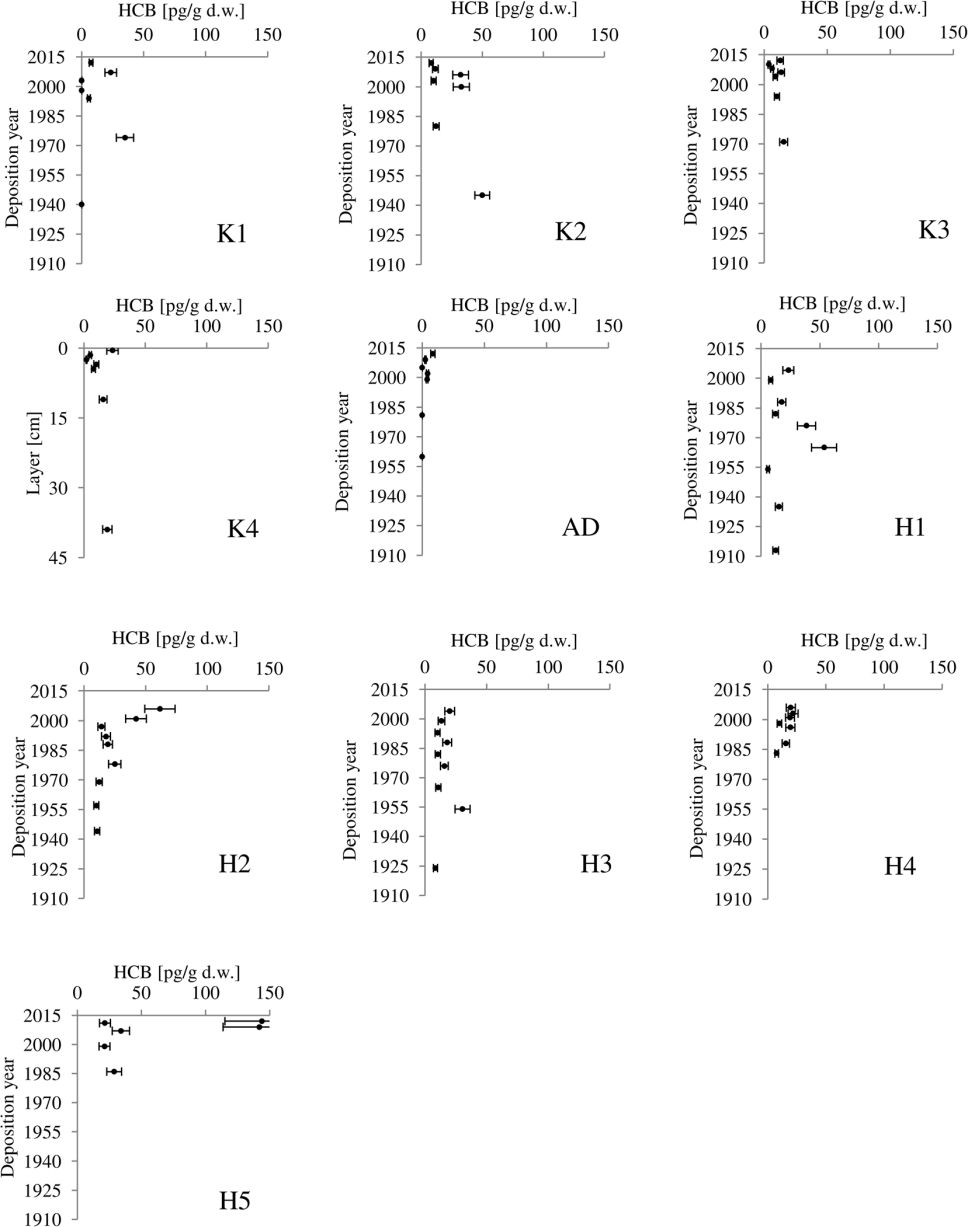
The HCB concentrations [pg/g d.w] measured in dated sediment cores collected from Kongsfjorden, Hornsund and Adventfjorden

The HCB concentrations ranged from below limit of quantification (6.86 pg/g d.w.) to 143.99 pg/g d.w. (Fig. 2). The detailed results on HCB are presented in the supplement material (Table S1). In Kongsfjorden, HCB concentrations varied from below limit of quantification (6.86 pg/g d.w.) to 50.01 pg/g d.w. At station H3, HCB concentration profile is generally constant and its concentration oscillated around 10 pg/g d.w. At other stations, some temporal variability in HCB concentrations can be noticed. At station K1 located in the outer fjord, the largest concentrations were measured at sediment layer dated for ~ 1975. At station K2, two HCB concentration peaks can be recognized one in deepest sampled sediment layer (dated for 1944–1945) and in sub-surface sediments (dated for 2000–2010) (Fig. 2). At station K4 located near glacier runoff, HCB concentration was similar to concentrations at other stations. Although it was impossible to determine sediment accumulation rate at this station, it was estimated for 6–7 cm/year based on other study (Trusel et al. 2010); thus, 45-cm-long sediment cores represent the time of 7–8 years. In Hornsund, concentrations of HCB ranged from limit of quantification (6.86 pg/g d.w.) to 143.99 pg/g d.w. The highest concentrations of HCB were measured in two surface sediment layers of the core collected at station H5 located near melting glacier. At station H1, the peak of HCB concentration reaching 54 pg/g d.w. was present in the sediment layer accumulated in late 1960s, while at station H3, the smaller peak (concentration of 30 pg/g d.w.) was present in layer dated for ~ 1955. Interestingly at station H2, HCB concentration was low until ~ 2000 and then started to increase. In Adventfjorden, concentrations of HCB ranged from below limit of quantification (6.86 pg/g d.w.) to 9 pg/g d.w. The vertical profile of HCB concentration in Adventfjorden was quite constant; it could be an effect of surface sediment mixing.

The HCB concentrations measured in this study were generally higher than concentrations (< 10 pg/g d.w.) detected recently in surface sediments of Kongsfjorden (Szczybelski et al. 2016). The historical study of HCB concentrations in surface sediments of Kongsfjorden reports higher HCB concentrations however (120–400 pg/g d.w.; Akvaplan-niva 1998). Our results are more similar to HCB concentrations detected in the Bering and Chuckchi Sea sediments and ranging from 40 to 130 pg/g d.w. (Strachan et al. 2001; Iwata et al., 1993) (Table 3). HCB concentrations detected in this study are lower than HCB concentrations of 2010 pg/g d.w. detected by Zaborska et al. (2011) and Szczybelski et al. (2016) in sediments from the Barents Sea. Concentrations of HCB in our study are also much lower than HCB detected in Northern Norway (reaching up to 6700 pg/g d.w.) (Dahle et al. 2000) (Table 3).

**Table 3:** HCB concentrations in sediments from different Arctic regions. Compound concentrations below the quantification limit are marked “b.d.”

Location		HCB concentration [pg/g d.w.]	References
Adventfjorden		b.d.–8.0	This study
Hornsund		6.8–140	
Kongsfjorden		b.d.–50	
		120–400	Akvaplan-niva (1998)
		b.d.–10	Szczybelski et al. (2016)
North Norway fjords		50–6700	Dahle et al. (2000)
North Russian fjords	Kola Bay	100–1500	
		90–1,350,000	Savinova et al. (2000)
	Guba Zapadnaya Litsa	150–2800	
	Guba Penchenga	280–1800	Dahle et al. (2000)
		280–1760	Savinov et al. (2003)
Barents Sea		b.d.–370	Szczybelski et al. (2016)
		b.d.–2010	Zaborska et al. (2011)
Bering Sea		100	Strachan et al. (2001)
		b.d.–2.7	Ma et al. (2015)
Bering Strait		5.4–97.2	
Norwegian Sea		5.5–10.0	
Arctic Ocean	Amundsen Basin	11.6	
	Canada Basin	b.d.–6.8	
Chukchi Sea		13.6–78.2	
		130	Strachan et al. (2001)
		35	Iwata et al. (1993)
		490	RAIPON/AMAP/GEF Project (2001)

The lowest concentrations of HCB were measured in Adventfjorden, suggesting that local anthropogenic activity is not a significant primary source of HCB and global transport processes are the major HCB transport pathways from lower latitudes. The highest concentrations of HCB were detected in sediment layers deposited recently in the inner Hornsund, Brepollen, in the vicinity of glacier outflow. At the same station, the Σ7 PCB and Σ12 PAH concentrations were also at their highest (Pouch et al. 2017). The high concentration of organic contaminants in the vicinity of the two glaciers may be connected to freshwater runoff from the glaciers. Substances accumulated over decades on the glacier surface have recently been discharged to the fjords in the course of intense glacier melting (Błaszczyk et al. 2013).

Although hydrophobic organic contaminants are expected to bind to organic matter and fine sediments (Hung et al. 2010; Sapota et al. 2009), in this study, no correlations were found between HCB concentrations and organic matter or pelite fraction content (*p* > 0.05, Table 4), reflecting its lower affinity for organic carbon and pelite fraction. This finding can suggest that scavenging of HCB from the water column is not controlled by suspended particulate matter and/or sediment grain size and organic matter content. Fjord sediments receive contaminants from very complex primary and secondary sources, e.g., the atmosphere, ocean currents, melting glaciers, and/or melting sea ice. Moreover, the importance of particular sources may vary seasonally as largest influx of glacier meltwaters and largest introduction of Atlantic waters occur during the summer. Additionally, in effect of summer sea ice cover reduction, HCB primary trapped is sea ice is transferred to seawater and the atmosphere (Grannas et al. 2013). It is thus possible that influence of regional conditions overwhelms the influence of suspended matter/sediment composition; nevertheless, some particular components of organic matter, e.g., black carbon content, may also play significant role in organic pollutant sorption (Bucheli et al. 2004; Staniszewska et al. 2011).

**Table 4:** The *R* coefficients and *p* values for the Spearman correlation between HCB concentrations and *M*_org_ content and fine fraction content in sediments from three Svalbard fjords

Fjord	*M*_org_ content (%)	Fine fraction (%)
HCB pg/g (Kongsfjorden)	− 0.05 (*p* > 0.05)	− 0.05 (*p* > 0.05)
HCB pg/g (Hornsund)	− 0.10 (*p* > 0.05)	− 0.20 (*p* > 0.05)
HCB pg/g (Adventfjorden)	0.71 (*p* > 0.05)	0.56 (*p* > 0.05)
HCB pg/g (all samples)	− 0.09 (*p* > 0.05)	0.04 (*p* > 0.05)

### HCB deposition rates

**Fig. 3:**
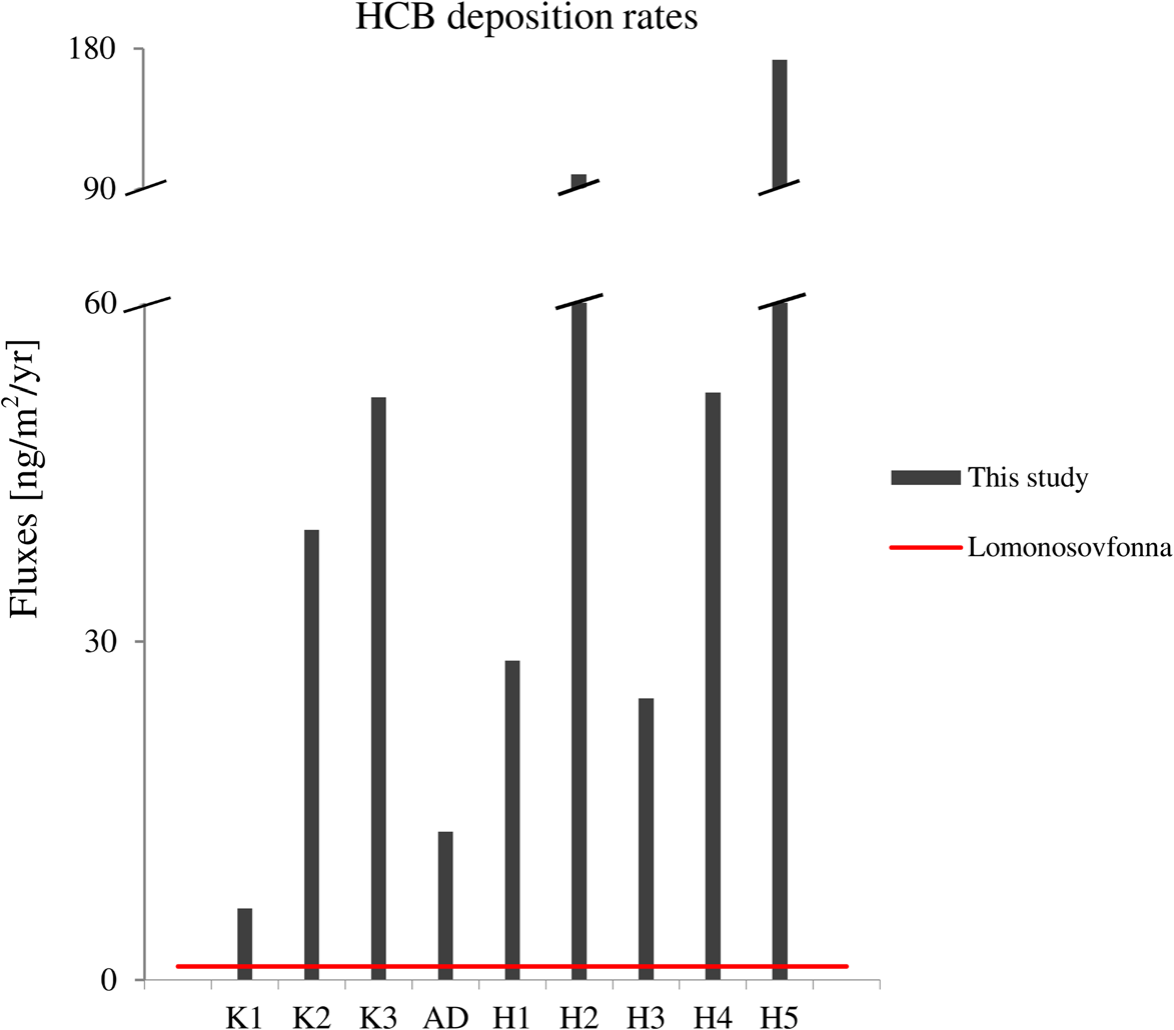
HCB deposition rates [ng/m^2^/yr] calculated for Kongsfjorden, Adventfjorden, and Hornsund (this study) in comparison to atmospheric HCB fluxes estimated for Lomonosovfonna (Svalbard) by Matthews et al. (2001)

Matthews (2001) estimated atmospheric HCB deposition rates for Svalbard region (Lomonosovfonna) to be 1.2 ng/m^2^/yr in 2000. The HCB deposition rates obtained in this study for the same time period were much higher than the aforementioned estimation. Calculated mean HCB accumulation rates ranged from 6.3 to 51.7 ng/m^2^/yr in Kongsfjorden, from 25.0 to 172.8 ng/m^2^/yr in Hornsund, and were of 13.1 ng/m^2^/yr in Adventfjorden (Fig. 3). This can suggest that direct deposition from the atmosphere is not the main factor affecting HCB levels in fjord sediments. The HCB deposition rates are very large in Brepollen (H5) and in inner Kongsfjorden (K3)—the regions influenced by glacial outflow. This can suggest that although in this marine environment some proportion of HCB can originate from oceanic circulation, the most important contaminant source is secondary discharge from melting glaciers.

### History of HCB deposition

**Fig. 4:**
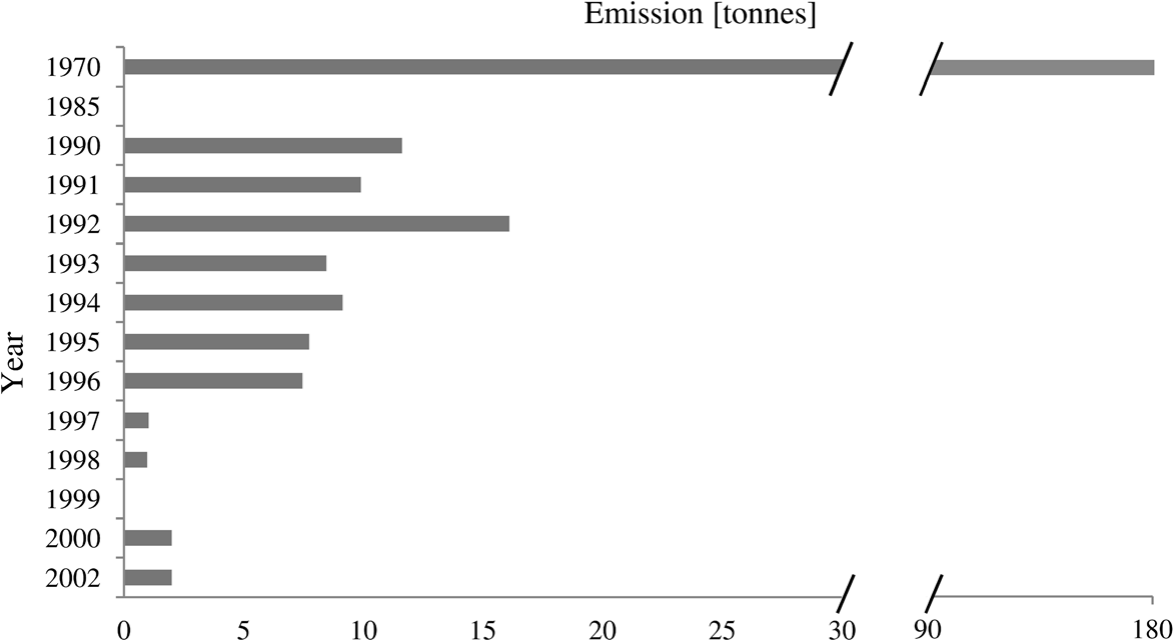
Emissions of HCB [tonnes] from Europe, according to data published by Barber et al. (2005) and Shatalov et al. (2001). Since 1970, HCB production has been restricted in most countries

HCB production and emission was largest during the 1950s and 1960s. HCB emission has been monitored since its production was restricted in most countries. Since 1970, emission of this contaminant has decreased significantly (Fig. 4). The history of HCB contamination revealed within this study does not completely mirror the global history of HCB emission. At some locations (H1, H3, K1, and K2), peaks of HCB were observed in sediment layers deposited from 1940 to 1970 during the largest use of HCB (Fig. 2). At other locations (H2, H5, K4), a recent increase in concentrations of HCB was observed, which could be caused by secondary emission of HCB, e.g., from melting of glaciers and permafrost.

**Fig. 5:**
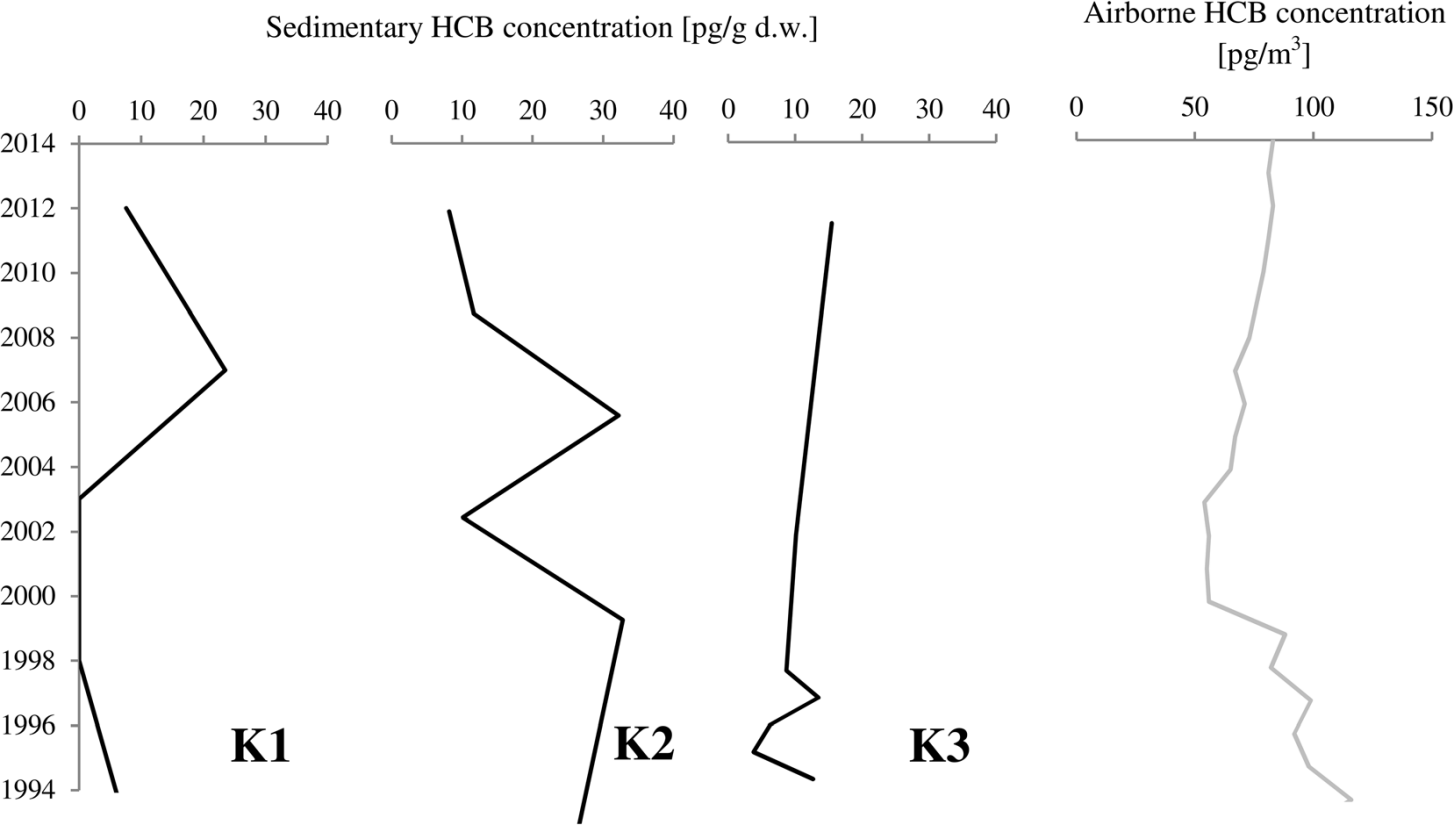
Comparison of sedimentary HCB concentrations [pg/g d.w.] at three stations in Kongsfjorden and airborne HCB concentration [pg/m^3^] measured in Ny-Ålesund, Kongsfjorden (NILU, 2016)

The airborne HCB concentration in Ny-Ålesund (Kongsfjorden) has been monitored since 1993 (http://www.nilu.no/projects/ccc/onlinedata/pops/index.html; NILU 2016). The mean HCB concentrations oscillated around 4.5 pg/g from 1993 to 1999 (Fig. 5). Then, from 2000 to 2004, the contaminant concentrations dropped to ~ 4.0 pg/g, but have been very slowly increasing since 2005. Although HCB concentrations are generally consistent over the last 20 years, they seasonally increase to 7 pg/m^3^. The comparison of airborne HCB concentrations with temporal concentrations of sedimentary HCB for the same time period in Kongsfjorden showed that such trends are quite similar for station K1 located at the Kongsfjorden entrance (Fig. 5). It is thus possible that in the outer fjords, located at the largest distance from the glaciers, the direct air deposition of HCB to the ocean surface is important, while for inner parts of the fjords, other processes mask the atmospheric HCB signal.

The differences between the calculated historical distribution and the emission history may be caused by variabilities in the air transport processes. In 1940–1970, the high accumulation of HCB in fjord sediments was caused by high global emission. After the restriction of HCB production in most countries, the accumulated contaminant concentrations are affected mostly by transport processes from secondary sources. Still high and/or increasing HCB concentrations in sediments deposited after 1970 coincided with a generally positive North Atlantic Oscillation (NAO) index (Macdonald et al. 2005).

Atmospheric circulation in the Arctic is driven by Arctic Oscillation (AO) that is correlated with the NAO. Contaminants are transported to the Arctic mainly during the winter and spring by three routes—from the Norwegian Sea, Eastern Europe/Siberia, and the Bering Sea, with the importance of each route depending on the NAO index (Macdonald et al. 2005). Concentrations of HCB in the air were found to correlate significantly (*p* < 0.05) with the NAO in the Great Lake region (Alert station) in the winter-spring season (Hung et al. 2005). Observed changes in atmospheric circulation, particularly the generally positive phase of the NAO/AO (Vavrus and Harrison 2003), result in more intense and extending farther north wind transport. That is why organic contaminants may be more effectively transported to Arctic regions.

Organic contaminants are removed from the atmosphere by wet and dry precipitation. Concentrations of HCB in snow collected on Bear Island ranged from 1.1 to 2 pg/dm^3^ (Evenset et al. 2002), while in the more contaminated Great Lakes, precipitation concentrations reaching up to 174 pg/dm^3^ were detected (Oliver and Nicol, 1982). Kongsfjorden and Hornsund receive similar annual precipitation of 450 mm (Førland et al. 2011; Błaszczyk et al. 2013), while precipitation in Adventfjorden is lower (~ 200 mm, Førland et al. 2011). Thus, the fact of lower measured HCB concentrations in Adventfjorden may be explained by the lower precipitation in this area compared to other studied fjords. The long-term observational series indicate an increase of precipitation in Svalbard region, and the projections indicate a further increase up to year 2100 (Førland et al. 2011). Schiedek et al. (2007) suggested that increase in precipitation will be accompanied with more effective “washing” of contaminants from the atmosphere. This effect may be notably observed in Svalbard.

### Environmental HCB half-lives in fjord sediments

The calculation of contaminant half-life in the environment has recently been used to estimate the time necessary for a 50% reduction in peak contaminant concentration due to emission reduction (Assefa et al. 2014; Sobek et al. 2015). In case of studied cores, the pronounced historical peaks of HCB were observed at only three stations, although additional sub-surface peaks of elevated HCB concentration were also observed (Fig. 2). At the other stations, there was no clear decreasing trend over time, and it was not possible to determine an environmental half-life for HCB. The calculated environmental half-lives of HCB in three fjord sediments (K1, K2, H1) ranged from 17 to 31 years (Table 5). The environmental HCB half-lives obtained in this study are similar to the half-lives of 29–32 years for PCDD/Fs, estimated by Assefa et al. (2014), for off-shore Baltic Sea sediments. They also agree with the half-lives of different PCBs reported by Sobek et al. (2015) for the central part of the Baltic Sea (14 ± 5 to 21 ± 12 years). The calculated half-lives confirm the finding of Assefa et al. (2014) that remote regions respond more slowly to contaminant emission reduction, as they receive contaminants both from direct influx from the atmosphere (primary source) combined with an influx of recycled contaminants previously deposited elsewhere (secondary source). In contrast, shore sediments located near populated coastal regions respond faster to contaminant emission restrictions (Sobek et al. 2015).

**Table 5:** Time of sediment layer deposition, peak and surface sediment concentration of HCB, and calculated HCB environmental half-life in the investigated sediment cores

Station	Deposition time	Peak conc. [pg/g d.w.]	Surface conc. [pg/g d.w.]	Half-life [years]
K1	1968–1978	34.99	7.57	17
K2	1940–1950	50.01	8.15	26
H1	1958–1972	53.55	23.17	31

### Sediment quality and ecotoxicological risk assessment

Generally, benthic organisms living at the sea bottom in the Arctic are often exposed to toxic concentrations of organic contaminants (AMAP 2004). Nevertheless, the concentrations of HCB measured in our study (max. 0.14 ng/g d.w.) can be assigned to the class I (background) of Norwegian environmental quality classification for sediments (0–0.5 ng/g; Bakke et al. 2010). Obtained concentrations were also well below the level of 17 ng/g that is calculated to be a predicted no effect HCB concentration for chronic exposure (PNEC_chronic_) for organisms living in sediments (SFT 2007a,b). The HCB concentrations measured in Svalbard fjords within other studies (AMAP 2004; Szczybelski et al. 2016) also did not exceed background concentration levels. Sediments containing HCB at concentrations which may cause toxic effects (class III) were found in northern Norway, while in Kola Bay, the concentrations of HCB were so high that they could cause severe toxic effects even during a short period of exposure (Dahle et al. 2000).

## Conclusions

The data presented in this study provide a baseline record of contamination by HCB in Svalbard fjords in present day and historic sediments (over the last 100 years). In our study, the measured HCB concentrations were generally low and ranged from below the limit of quantification (6.86 pg/g d.w.) to 144 pg/g d.w. However, the historical profiles of HCB concentrations show an evident increase of contaminants in the surface sediments at some of the investigated locations, suggesting that although HCB production is banned in most countries, still a lot of this compound can reach arctic marine environment. The measured HCB deposition rates (6–173 ng/m^2^/yr) were much larger that the estimated airborne HCB flux suggests that direct flux from the atmosphere is not the main source of HCB for fjord sediments. The results suggest that HCB fluxes are largest close to glacier outflows, indicating a prevailing role of glacier melting in distribution of HCB within fjords. Although HCB concentrations are still relatively low, intense glacier and permafrost melting could lead to an increase of HCB concentrations. This can lead to a conclusion that recent global changes have caused an increase in the strength of HCB discharge to the Arctic fjords from primary and secondary sources. Nevertheless, the highest concentrations of HCB measured in our study did not exceed background concentration levels, and thus, a negative effect on benthic organisms is not expected.

## Electronic supplementary material

Fig S1 (DOCX 49 kb)

Table S1 (DOCX 21 kb)
