# Supplementary material for: The history of hexachlorobenzene accumulation in Svalbard fjords
Source: Environ Monit Assess. 2018 May 24;190(6):360. doi: 10.1007/s10661-018-6722-3 (PMC5968051; doi:10.1007/s10661-018-6722-3)
Supplement: Supplementary file 1 — (DOCX 49 kb) [file 10661_2018_6722_MOESM1_ESM.docx]

Fig. S1. The activity concentrations of ^210^Pb_tot_ in sediment cores collected from Kongsfjorden, Adventfjorden and Hornsund. The results obtained for stations K1, K2, K3, K4, AD and H5 were presented elsewhere (Zaborska et al., 2017) while results obtained for stations H1, H2, H3 and H4 are presented in this study.
