# Supplementary material for: The history of hexachlorobenzene accumulation in Svalbard fjords
Source: Environ Monit Assess. 2018 May 24;190(6):360. doi: 10.1007/s10661-018-6722-3 (PMC5968051; doi:10.1007/s10661-018-6722-3)
Supplement: Supplementary file 2 — (DOCX 21 kb) [file 10661_2018_6722_MOESM2_ESM.docx]

Tab. S1. Concentrations of HCB in sediments cores collected in Kongsfjorden, Adventfjorden and Hornsund.

| Station | Layer [cm] | Concentration [pg/g d.w.] | |
| --- | --- | --- | --- |
|  |  | $\bar{x}$ | SD |
| K1 | 0 – 1 | 7.57 | 1.51 |
| K1 | 1 – 2 | 23.50 | 4.70 |
| K1 | 2 – 3 | bd | bd |
| K1 | 3 – 4 | bd | bd |
| K1 | 4 – 5 | 5.89 | 1.18 |
| K1 | 10 – 12 | 34.99 | 7.00 |
| K1 | 18 – 20 | bd | bd |
| K2 | 0 – 1 | 8.15 | 1.63 |
| K2 | 1 – 2 | 11.61 | 2.32 |
| K2 | 2 – 3 | 32.20 | 6.44 |
| K2 | 3 – 4 | 10.13 | 2.03 |
| K2 | 4 – 5 | 32.81 | 6.56 |
| K2 | 10 – 12 | 12.22 | 2.44 |
| K2 | 24 – 26 | 50.01 | bd |
| K3 | 0 – 1 | 12.66 | 2.53 |
| K3 | 1 – 2 | 3.80 | 0.76 |
| K3 | 2 – 3 | 6.25 | 1.25 |
| K3 | 3 – 4 | 13.45 | 2.69 |
| K3 | 4 – 5 | 8.72 | 1.74 |
| K3 | 10 – 12 | 10.12 | 2.02 |
| K3 | 26 – 28 | 15.44 | 3.09 |
| K4 | 0 – 1 | 23.23 | 4.65 |
| K4 | 1 – 2 | 5.06 | 1.01 |
| K4 | 2 – 3 | 2.02 | 0.40 |
| K4 | 3 – 4 | 10.10 | 2.02 |
| K4 | 4 – 5 | 7.76 | 1.55 |
| K4 | 10 – 12 | 15.62 | 3.12 |
| K4 | 38 – 40 | 19.01 | 3.80 |
| AD | 0 – 1 | 8.64 | 1.73 |
| AD | 1 – 2 | 2.61 | 0.52 |
| AD | 2 – 3 | bd | bd |
| AD | 3 – 4 | 4.29 | 0.86 |
| AD | 4 – 5 | 3.90 | 0.78 |
| AD | 10 – 12 | bd | bd |
| AD | 18 – 20 | bd | bd |
| H1 | 0 – 1 | 23.17 | 4.63 |
| H1 | 1 – 2 | 8.18 | 1.64 |
| H1 | 3 – 4 | 17.47 | 3.49 |
| H1 | 4 – 5 | 12.26 | 2.45 |
| H1 | 5 – 6 | 38.59 | 7.72 |
| H1 | 7 – 8 | 53.55 | 10.71 |
| H1 | 9 – 10 | 5.90 | 1.18 |
| H1 | 12 – 14 | 15.18 | 3.04 |
| H1 | 16 – 18 | 12.45 | 2.49 |
| H2 | 0 – 1 | 61.62 | 12.32 |
| H2 | 1 – 2 | 42.23 | 8.45 |
| H2 | 2 – 3 | 14.17 | 2.83 |
| H2 | 3 – 4 | 17.79 | 3.56 |
| H2 | 4 – 5 | 19.30 | 3.86 |
| H2 | 5 – 6 | 24.96 | 4.99 |
| H2 | 7 – 8 | 12.27 | 2.45 |
| H2 | 9 – 10 | 10.02 | 2.00 |
| H2 | 14 – 16 | 10.69 | 2.14 |
| H3 | 0 – 1 | 19.94 | 3.99 |
| H3 | 1 – 2 | 13.39 | 2.68 |
| H3 | 2 – 3 | 10.20 | 2.04 |
| H3 | 3 – 4 | 17.95 | 3.59 |
| H3 | 4 – 5 | 10.44 | 2.09 |
| H3 | 5 – 6 | 15.73 | 3.15 |
| H3 | 7 – 8 | 10.81 | 2.16 |
| H3 | 9 – 10 | 30.40 | 6.08 |
| H3 | 14 – 16 | 8.45 | 1.69 |
| H4 | 0 – 1 | 19.72 | 3.94 |
| H4 | 1 – 2 | 21.66 | 4.33 |
| H4 | 2 – 3 | 19.07 | 3.81 |
| H4 | 3 – 4 | 9.96 | 1.99 |
| H4 | 4 – 5 | 19.37 | 3.87 |
| H4 | 7 – 8 | 15.48 | 3.10 |
| H4 | 9 – 10 | 7.57 | 1.51 |
| H5 | 0 – 1 | 143.99 | 28.80 |
| H5 | 1 – 2 | 21.55 | 4.31 |
| H5 | 2 – 3 | 142.07 | 28.41 |
| H5 | 3 – 4 | 34.01 | 6.80 |
| H5 | 10 – 12 | 21.28 | 4.26 |
| H5 | 22 – 24 | 28.77 | 5.75 |
